# Milk fat globule membrane in early-life nutrition: composition, production, and biological effects on infant immune maturation, intestinal development, neurocognitive function, and growth

**DOI:** 10.3389/fnut.2026.1851487

**Published:** 2026-06-18

**Authors:** Emine Kocyigit, Elif Çelik, Özge Cemali, Oya Berkay Karaca, Bence Raposa, Duygu Ağagündüz

**Affiliations:** 1 Faculty of Health Sciences, Department of Nutrition and Dietetics, Ordu University, Ordu, Türkiye; 2 Faculty of Health Sciences, Department of Nutrition and Dietetics, Süleyman Demirel University, Isparta, Türkiye; 3 Faculty of Health Sciences, Department of Nutrition and Dietetics, Trakya University, Edirne, Türkiye; 4 Faculty of Fine Arts, Department of Gastronomy and Culinary Arts, Cukurova University, Adana, Türkiye; 5 Institute of Basics of Health Sciences, Midwifery and Health Visiting, Faculty of Health Sciences, University of Pécs, Pécs, Hungary; 6 Faculty of Health Sciences, Department of Nutrition and Dietetics, Gazi University, Ankara, Türkiye

**Keywords:** growth, health, infant, microbiome, milk fat globule membrane, neurocognitive

## Abstract

The milk fat globule membrane (MFGM) is a trilayered structure encasing fat globules in mammalian milk, primarily composed of phospholipids, glycoproteins, and bioactive molecules. Recent research indicates that MFGM plays a fundamental role in early-life health, particularly through its effects on gut microbiota development and immune system maturation. In the initial months of life, the infant’s gut microbiome undergoes rapid colonization, essential for immunological tolerance, metabolic programming, and pathogen defense. Numerous studies have shown that MFGM components, including sphingomyelin, gangliosides, and glycoproteins, exhibit prebiotic-like effects by facilitating the proliferation of beneficial bacteria, such as *Bifidobacterium* and *Lactobacillus* species. Furthermore, MFGM supplementation in infant formulas has been linked to microbiota profiles that more closely resemble those of breastfed infants, enhanced gastrointestinal function, cognitive development, and a decreased incidence of diseases. This review clarifies how MFGM influences gut microbiota regulation, including increased barrier function, anti-inflammatory properties, and pathogen defense. Moreover, it highlights the possibility of MFGM-enriched dietary approaches to enhance proper gut colonization and promote long-term infant health, particularly in formula-fed populations. Although the results are encouraging, more longitudinal and mechanistic investigations are necessary to comprehensively clarify the influence of MFGM on the gut microbiome and the microbiota-mediated health benefits for growth and development.

## 1 Introduction

The early years of life are a crucial time for organ development and metabolism, and they may also influence a person’s vulnerability to subsequent physiological or stress-related conditions (1). The development of the infant gastrointestinal tract, encompassing the growth of gut microbiota and the maturation of the immune system, has significant long-term implications for health (2). During and immediately following birth, the infant’s gut microbiome experiences rapid colonization, a phenomenon that aids in immunological tolerance, metabolic control, and pathogen defense. Multiple factors influence the composition of the infant gut microbiota, including mode of delivery, environmental exposures, antibiotic use, and dietary patterns, with diet representing one of the most significant determinants (3, 4). Microbial dysbiosis in early life has been linked to a heightened risk of illnesses like allergies and type 1 diabetes (3, 4). Consequently, understanding the determinants of early microbiota development and strategies that support its healthy establishment is essential for enhancing child health outcomes (5, 6).

Human milk provides neonates with essential nutrients (proteins, carbohydrates, long-chain fatty acids, vitamins, minerals, etc.), immunological protection (including antibodies and oligosaccharides), and support for gastrointestinal health—each of which is critical for normal growth and development (7, 8). The MFGM has attracted increasing interest due to its intricate structure and bioactive constituents. The MFGM is rich in phospholipids, sphingolipids, cholesterol, and glycoproteins, which may play roles in immunological modulation, gut barrier integrity, and neurodevelopment (7, 9, 10). In contrast to human milk, commercial infant formulas frequently lack the natural MFGM structure due to processing methods such as fat separation and homogenization. Consequently, there has been an increased interest in integrating bovine-derived MFGM into infant formula to more closely replicate the structural and functional characteristics of human milk (11–13). Recent studies indicate that infant formulas containing MFGM-coated lipid droplets may enhance lipid digestion and diminish metabolic disparities between formula-fed and breastfed infants (14, 15).

Over the past decade, MFGM has gained prominence in health research as a uniquely bioactive membrane rich in polar lipids and glycoproteins that are largely underrepresented in standard formulas (16). Experimental and clinical studies have indicated that MFGM supplementation may enhance neurodevelopment, alter gut microbiota composition, and provide immune protection during early life. Interest in this component has accelerated as preclinical work showed antiviral and barrier-protective actions (e.g., inhibition of rotavirus binding to intestinal cells and mitigation of mucosal injury), while clinical evidence—including recent randomized trials and a 2024 meta-analysis—has suggested potential benefits for infant neurocognitive outcomes (17–19). Contemporary reviews also highlight potential signals of reduced infection risk and the role of MFGM as an emerging nutraceutical in early-life nutrition. Collectively, these foundational observations justify a focused examination of MFGM composition to understand structure–function relationships and its translational potential (20, 21).

Besides its nutritional roles, MFGM has recently been investigated as a possible matrix for the stability and delivery of probiotics (22–24). The interactions between MFGM components and probiotic bacteria may improve bacterial viability and promote colonization in the gastrointestinal tract (25, 26). These characteristics indicate that MFGM may contribute to both infant nutrition and the advancement of functional foods and novel delivery strategies for bioactive compounds.

A comprehensive synthesis of the current evidence is therefore needed, as the evidence is expanding but still limited. This study reviews the structure and bioactive content of MFGM. It also assesses its possible effects on infant health, focusing on gut microbiota development, immune maturation, neurodevelopment, and growth. The study examines the growing use of MFGM as a probiotic carrier and highlights areas for future research.

## 2 Literature search strategy

A systematic literature search was performed in PubMed, Web of Science, and Scopus using a consistent, reproducible protocol. Predetermined keyword combinations focused on core research areas: milk fat globule membrane and infant/neonate health. Keywords included “milk fat globule membrane,” “MFGM,” “infant nutrition,” “infant formula,” “gut microbiota,” “immune development,” “neurodevelopment,” “cognitive development,” “growth,” “probiotics,” and “clinical study.” Boolean operators (AND/OR) were applied to combine these search terms. Original research papers, clinical trials, preclinical studies, and relevant reviews examining MFGM content, its function in infant/neonate feeding, and its effects on child health were evaluated. Studies analyzing MFGM’s influence on gut microbiota, immune development, neurodevelopment, growth metrics, and probiotic strategies were eligible. Studies unrelated to MFGM or infant health, as well as those failing to link these variables to gut microbiota, immune development, neurodevelopment, growth, or probiotics were excluded. Selected papers were screened by title and abstract, followed by a full review if appropriate. Inclusion depended on topic relevance, research design, and meeting the review’s goals. This protocol strengthened transparency and broadened the scientific scope of the review.

## 3 Structure and composition of milk fat globule membrane

A comprehensive understanding of the mechanisms underlying membrane formation is crucial for interpreting the complex architecture of the milk fat globule membrane (MFGM) and its potential biological implications (21, 27). The MFGM, which encapsulates milk fat globules (MFGs) in mammalian milk, has a nanoscale trilayer structure that stabilizes and protects MFGs. These globules typically range in diameter from approximately 0.1 to 15 μm, averaging about 4 μm (28). This membrane, formed during the secretion of lipid droplets by mammary epithelial cells, acts as a barrier that protects the lipid core and regulates molecular interactions-functions that are integrated with lipid synthesis, intracellular trafficking, and membrane remodeling. Neutral lipids accumulate within the endoplasmic reticulum (ER) membrane. This accumulation leads to the formation of cytoplasmic lipid droplets, whose phospholipid monolayer, derived from the ER, provides a surface that separates the hydrophobic lipid core from the aqueous cytoplasm, thus enabling proper lipid storage and mobilization (29, 30). As lipid droplets migrate toward the apical surface of mammary epithelial cells, they become encapsulated by the apical plasma membrane. This process results in a characteristic trilaminar membrane structure, composed of an endoplasmic reticulum-derived monolayer and a plasma membrane bilayer, that both shields the lipid contents from the external environment and provides a functional interface regulating secretion (31).

The MFGM originates during lipid synthesis and fat globule secretion within mammary epithelial cells. It is derived from three distinct sources: the apical plasma membrane, the endoplasmic reticulum (ER), and post-Golgi vesicular structures of mammary epithelial cells (16). Small lipid droplets (< 0.5 μm in diameter) initially form within the endoplasmic reticulum, where neutral lipids accumulate between the ER bilayer leaflets and are subsequently released into the cytosol as cytoplasmic lipid droplets surrounded by a phospholipid monolayer. ER-associated proteins contribute to the stabilization and maturation of lipid droplets before their transport toward the apical plasma membrane. During the early stages of lipid droplet formation, a fixed spacing of approximately 10–20 nm is observed between lipid droplets and the apical plasma membrane. This space is associated with the electron-dense inner leaflet of the apical plasma membrane and associated phospholipids (PLs), which constitute the core components of the MFGM bilayer (16, 28, 32).

Structurally, the MFGM is composed predominantly of lipids (~30–75%) and proteins (~25–70%), along with smaller amounts of carbohydrates (mainly glyco-conjugates) and trace RNA, and it contributes to stabilizing the fat globule as a natural oil-in-water emulsion (33, 34). This membrane stabilizes milk fat globules within a natural oil-in-water emulsion. Enriched with phospholipids, sphingolipids, cholesterol, and membrane-associated glycoproteins, it serves as a biologically active interface that facilitates selective molecular interactions and enhances functional stability in milk. Its composition may vary based on several factors, including species physiology, extraction and purification methods, milk processing, and analytical techniques used to characterize molecular components (35, 36).

Recent research suggests that infant formulas containing large, phospholipid-coated lipid droplets,engineered to mimic the natural MFGM, may produce beneficial effects on gastrointestinal and neurodevelopmental outcomes (21, 37). A randomized trial reported that the so-called “concept formula” accelerated gut microbiota maturation, promoting butyrate-producing taxa and enhancing metabolomic indicators, while reducing excess adiposity during the first year of life (37). Numerous randomized trials have examined the impact of bovine MFGM-enriched formulas on infant health outcomes. One study reported improved neurodevelopmental outcomes up to 6.5 years of age (38), while the COGNIS follow-up study demonstrated enhanced cognitive performance and alterations in brain structure at six years among children who consumed MFGM-supplemented formula during infancy (39). Recent reviews systematically highlight the nutritional and functional reasons for adding MFGM to infant formulas, as this approach may allow formulas to more closely resemble the structure and bioactivity of human milk. Because key MFGM components exhibit distinct bioactive effects, replicating these features in infant formula may support developmental outcomes similar to those observed in human milk (34, 40). It is important to acknowledge that several intervention studies assess MFGM with other bioactive components, thereby complicating the assignment of observed health benefits exclusively to MFGM supplementation.

**Figure 1:**
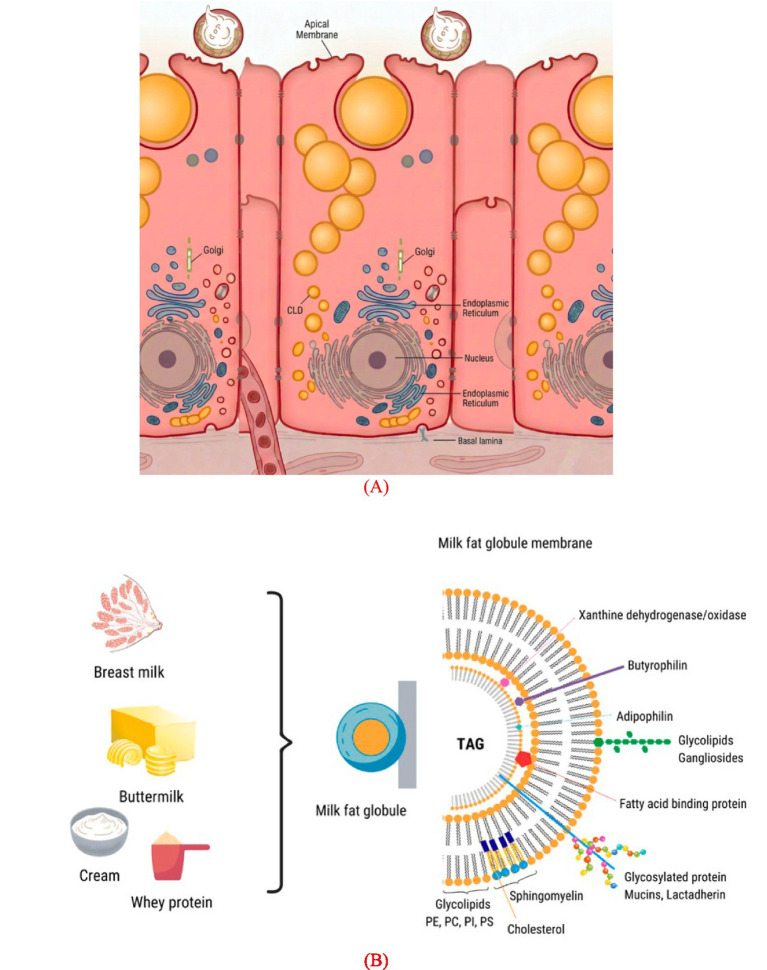
Biosynthesis and secretion of milk fat globules in mammary epithelial cells and structural composition of the milk fat globule membrane. **(A)** Milk fat globule biosynthesis in mammary epithelial cells. Cytoplasmic lipid droplets (CLDs) are formed from triacylglycerols synthesized in the endoplasmic reticulum. These droplets migrate toward the apical plasma membrane. When secreted, the droplet is surrounded by the apical membrane and released into the milk lumen as a mature milk fat globule. This process helps form the trilayer of the milk fat globule membrane. The trilayer includes an endoplasmic reticulum-derived inner phospholipid monolayer and an apical membrane-derived outer phospholipid bilayer (27). **(B)** Breast milk, buttermilk, cream, and whey-derived fractions represent the primary biological and industrial sources of MFGM. The schematic on the right illustrates the triacylglycerol (TAG) core surrounded by the trilayered MFGM, which is enriched in polar lipids (phosphatidylethanolamine [PE], phosphatidylcholine [PC], phosphatidylinositol [PI], phosphatidylserine [PS], and sphingomyelin), glycolipids, gangliosides, cholesterol, and membrane-specific proteins (e.g., butyrophilin, adipophilin, xanthine dehydrogenase/oxidase, mucins, lactadherin, fatty acid binding protein). These components confer the structural and functional properties of MFGM that underlie its proposed biological effects [Adapted from O’Callaghan et al. (85), created using Canva].

Significant knowledge gaps remain. Experimental and clinical evidence suggest MFGM supplementation may be beneficial (41, 42). However, the effects of specific MFGM components on infant outcomes are unclear. Polar lipids may influence neurodevelopment by affecting cell signaling and synaptogenesis. Sphingolipids could alter gut microbiota and help maintain intestinal barriers. Membrane-associated proteins may aid immune maturation by modulating immune responses (43–45). However, the underlying mechanisms remain insufficiently characterized. Most clinical studies test MFGM with other bioactive ingredients in formulas, making it difficult to isolate MFGM’s effects. Comprehensive evidence synthesis is needed to clarify structure–function relationships and guide translational research priorities. The biosynthesis and secretion of MFGs in mammary epithelial cells, together with the structural composition of the MFGM, are shown in Figure 1.

### 3.1 Milk fat globule membrane lipids

The primary function of MFGM is to provide fatty acids to the infant (36). Triacylglycerols (TAGs) are essential lipids for growth and development and comprise roughly half of an infant’s energy needs and 98% of total milk fat. TAGs are the principal components of the MFG. TAGs are secreted as lipid globules in the mammary gland, surrounded by the MFGM (46). The two types of lipids found in the MFGM are polar and neutral. Polar lipids are composed of glycerophospholipids, including phosphatidylcholine (PC), phosphatidylethanolamine (PE), phosphatidylinositol (PI), and phosphatidylserine (PS), sphingophospholipids, such as sphingomyelin (SM), and glycosphingolipids, including gangliosides, cerebrosides, ceramides, and sphingosine. The predominant polar lipids in the human MFGM are SM (27–43%), followed by PC (14–38%), and PE (6–36%). PS and PI constitute 5–6% of the total, whereas ganglioside levels are relatively low (47, 48). Reports have suggested that neutral lipids may constitute around 56% of the MFGM fraction; however, these values often reflect inadequately separated preparations in which core triacylglycerols contaminated the membrane fraction. In the native milk fat globule structure, roughly 95–98% of neutral lipids are sequestered inside the triglyceride-rich core and were not regarded as components of the MFGM (49). Small amounts of diacylglycerol (DAG), monoacylglycerol (MAG), free fatty acids (FFAs), and sterols can range from 0.3 to 2%. Acylglycerols may be detected in MFGM preparations due to contamination occurring during isolation (21, 49). Cholesterol, an important constituent of the cell membrane, along with cholesterol esters, constitutes anadditional component of the neutral lipid component (50).

Lipids in the MFGM facilitate the development of the nervous system and cognitive function (21, 51). Polar lipids, including PLs, gangliosides, and SM, are crucial for the development of the brain and nervous system and for the functioning of the gut and immunity (52–55). SM has been reported to support language development in infants and mitigate memory deterioration; however, there is no direct clinical evidence that SM supplementation improves outcomes in Alzheimer’s disease (56, 57). It has been proposed that cholesterol absorption is reduced in the presence of sphingomyelin because of its strong affinity for cholesterol and its capacity to impede micellar solubilization. Sphingomyelin has been shown to decreas the thermodynamic activity of cholesterol monomers and downregulate NPC1L1 expression in enterocytes, thereby restricting intestinal absorption (58). In obese mouse models, dietary sphingomyelin supplementation decreased intestinal cholesterol absorption, increased fecal lipid excretion, and mitigated hepatic lipid accumulation (59).

### 3.2 Milk fat globule membrane proteins

MFGM proteins comprise over 100 protein species ranging from 13 to 240 kDa, constitute 1% of the entire globule mass (60), were initially separated using two-dimensional electrophoresis in the late 1990s, with Goldfarb (1997) delivering the earliest systematic protein map via 2-DE. However, proteomic analyses have since progressed considerably: contemporary approaches now frequently employ high-resolution LC–MS/MS, coupled with label-free quantification and glycoproteomic workflows, enabling more comprehensive and quantitative profiling of MFGM protein composition across species and lactational stages (34, 61). MFGM proteins have an asymmetric distribution; proteins embedded within or on both sides of the MFGM are termed integral proteins, while those affixed to the outer surface of the MFGM are referred to as peripheral proteins. Furthermore, some proteins have a weak association with the MFGM, which is crucial for maintaining its physiological functionality (36). Alongside prevalent MFGM proteins, α-lactalbumin, lysozyme, β-casein, clusterin, lactoferrin, immunoglobulins, tenascin, apolipoproteins, and fatty acid synthase have been consistently detected in human MFGM across many proteomic analyses (62, 63).

Membrane proteins are present in both glycosylated and non-glycosylated forms. Glycosylated proteins provide essential functions in cell interaction, localization, immunity, growth, and bacterial interactions. This indicates that MFGM facilitates other biological processes in an infant’s gut beyond energy provision (64, 65). Prominent MFGM proteins include mucin 1 (MUC1), xanthine dehydrogenase/oxidase (XDH/XO), cluster of differentiation 36 (CD36), butyrophilin (BTN), adipophilin (ADPH), periodic acid Schiff 6/7 or lactadherin (PAS 6/7), proteose peptone 3 (PP3), and fatty acid binding protein (FABP). Proteins within the outer membrane of mammary epithelial cells are often glycosylated, except for FABP and ADPH (66, 67). Human MFGM contains carboxyl ester lipase and FABP, whereas bovine MFGM contains glycosylation-associated cell adhesion molecule 1 (GlyCAM1) (12). Bovine milk also contains FABPs, which were suggested to help in the transport of fatty acids across the MFGM (68). However, instead of being a lipid-binding factor, GlyCAM1 was shown to be an immune-related adhesion protein in bovine MFGM. Therefore, GlyCAM1 should not be regarded as functionally analogous to FABPs found in human milk, as its biological role is more closely linked to lymphocyte adhesion and immune signaling (69).

MUCs, including MUC1 and MUC4, represent a category of proteins prevalent in human MFGM (70). MUC1, characterized by high glycosylation, traverses the outer bilayer membrane and facilitates intercellular interaction. It acts as a barrier between microorganisms and connective tissue, safeguarding the gastrointestinal tract of infants from harmful pathogens and exhibiting resistance to digestion (71, 72). BTN, a significant protein found in bovine MFGM, is characterized as a glycosylated transmembrane protein that is integrated into the outer bilayer membrane, similar to MUC1. BTNs belong to the immunoglobulin superfamily, with BTN subfamily one member A1 (BTN1A1) being the specific variant present in human MFGM (34, 73, 74). Han et al. discovered that the knockdown of BTN1A1 in bovine mammary epithelial cells decreased both the size and phospholipid content in lipid droplets. The findings of this study demonstrate that BTN1A1 plays an essential role in regulating lipid droplet production through a mechanism associated with membrane phospholipid content (75). In the study by Pan et al. (15), it was found that adding MFGM before homogenization and milk proteins afterward resulted in a lipid droplet interfacial composition more similar to that of human milk, significantly enhancing lipid digestion by increasing free fatty acid release (76). Zhao et al. (77) found that the interaction between whey protein, the main protein in infant formula, and milk fat globule membrane phospholipids significantly increased the physical stability and fat digestion performance of the emulsion by forming a dense and stable structure at the interface of fat globules (77). A significant improvement in the degree of lipolysis was observed, particularly at an MFGM phospholipid: whey protein ratio of 5:10, with smaller particle size, higher surface charge, and increased free fatty acid release (77).

ADPH (also called perilipin 2) and FABP, which have a high affinity for triglycerides, are included as part of the non-glycosylated MFGM proteins within the inner phospholipid membrane (78). By binding to fatty acids, FABP can control fatty acid solubilization, storage, and transportation across organelles and membranes in cells (79). ADPH controls lipolysis by managing protein access to the lipid droplet surface (80). FABP prevents the adhesion of *Escherichia coli* to intestinal epithelial cells (*in vitro*) (81). XDH/XO is a redox enzyme in the intermembrane gap between the monolayer and bilayer, comprising 12% of bovine MFGM proteins (82). XDH/XO forms a tripartite structure with BTN and ADPH, enabling the integration of the apical cell membrane with lipid globules. XDH/XO enhances the antibacterial defense of the gastrointestinal tract through interactions with multiple substrates. Proteomic analyses revealed that XDH/XO is among the most prevalent proteins consistently identified in bovine MFGM, frequently reported with BTN and ADPH, suggesting a structural function in preserving membrane integrity rather than a direct necessity for milk fat secretion (34, 65).

Alongside the main MFGM proteins, MFGM comprises several proteins, including carbonic anhydrase, alkaline phosphatase, lactoferrin, osteopontin, lysozyme, and several enzymes present in low amounts. These proteins serve several functions in neonatal health, including acid neutralization, antimicrobial activity, growth promotion, regulation of innate immunity, and protection against digestion (50, 73).

### 3.3 Milk fat globule membrane carbohydrates

MFGM carbohydrates are conjugated with proteins and lipids to form glycoproteins or glycolipids, respectively. Glycolipids are categorized into two main types: neutral glycolipids and acidic glycolipids, which include gangliosides. Neutral glycolipids comprise several carbohydrate residues linked to ceramide within the lipid structure, such as glucosylceramide, lactosylceramide, and galactosylceramide. Acidic glycolipids contain oligosaccharides linked to ceramide and include at least one sialic acid residue connected through glycosidic bonds. MUC, BTN, CD36, and PAS 6/7, identified among the proteins in the MFGM, are classified as glycoproteins (83, 84).

## 4 Advanced strategies for the purification, enrichment, and production of milk fat globule membrane

Scientific and technological advancements have facilitated the extraction and purification of MFGM for diverse applications in nutrition, pharmaceuticals, cosmetics, and functional food systems. Recovering intact MFGM fractions may help preserve the biological properties of human milk and advance infant nutrition.

MFGM is present in fresh milk and cream, but buttermilk, the by-product of butter manufacturing, is considered the primary industrial source of MFGM constituents (85). Mechanical stressors such as agitation, phase transition, food processing, cooling, and heating affect MFGM structure and composition. These processing conditions may compromise the natural membrane structure, thus modifying the physicochemical and biological characteristics of MFGM-derived components (33, 86). Therefore, enhancing fractionation and purification methods is crucial to maintaining the functional integrity of MFGM.

Microfiltration, ultrafiltration, and diafiltration efficiently concentrate MFGM fractions from dairy by-products. Compared with traditional processing procedures, these techniques enhance the production of phospholipids and membrane-associated proteins while minimizing contamination from non-MFGM proteins (87, 88). Enzymatic treatments can enhance phospholipid recovery; proteolytic enzymes such as pepsin and trypsin hydrolyze caseins and whey proteins, thereby enabling the concentration of phospholipid-rich MFGM fractions by subsequent membrane filtration (84, 89). Importantly, various extraction and purification methods may uniquely influence the structural integrity of MFGM. Membrane filtration methods often maintain natural membrane fragments, whereas solvent extraction and rigorous heat processing might compromise protein-lipid complexes, thereby diminishing biological functionality.

Two dairy-based products contain MFGM components: phospholipid extracts and MFGM-enriched fractions. Phospholipid extracts are typically generated by solvent extraction and are frequently utilized in cosmetic and dermatological formulations, whereas MFGM-enriched components derived from physical separation techniques are predominantly designed for nutritional uses (8, 90). Commercially available MFGM-fortified products include Lacprodan® MFGM-10 (Arla Foods), Vivinal® MFGM (FrieslandCampina Ingredients, Amersfoort, The Netherlands), NZMP SureStart™ Lipid 100, SureStart™ 70, and MFGM Complex Lipids (Fonterra). Some nutraceutical companies are adding MFGM to their omega-3 supplements, which are associated with various health benefits (48, 91). The central concern is whether separated MFGM fractions still preserve the supramolecular structure of the original membrane. Processing steps, including homogenization, heat treatment, and solvent-based extraction, are recognized to modify lipid and protein orientation, thereby compromising the stability of protein-lipid complexes and lowering bioactivity (16, 92). The structural modifications may partially elucidate the diversity observed in intervention studies using bovine-derived MFGM components, underscoring the importance of processing conditions in interpreting biological results.

Conventional infant formulas often include minimal intact MFGM due to procedures such as fat removal, homogenization, and drying; hence, extensive research has concentrated on reintroducing bovine-derived MFGM fractions to more accurately approximate the structural and functional characteristics of human milk (8, 16). Although clinical and preclinical studies have demonstrated the benefits of MFGM supplementation (9, 41), there is no globally standardized method for fortifying infant formula with MFGM-derived bioactive components.

### 4.1 Milk fat globule membrane as a novel encapsulation matrix for probiotics

MFGM is studied for its nutritional value in infant formula, especially for its ability to support probiotic survival and gut colonization (93). Encapsulation denotes the integration of bioactive compounds into protective matrices that improve their stability, viability, and controlled release during digestion (94–96). MFGM, due to its amphiphilic structure and intricate composition of phospholipids, glycoproteins, and glycolipids, has recently been investigated as a natural encapsulation matrix for probiotic microorganisms.

Interactions between MFGM components and probiotic bacteria may enhance bacterial adhesion and viability in the gastrointestinal environment. The external surface of milk fat globules is mostly hydrophilic, facilitating the adhesion of probiotic bacteria via interactions with phospholipids, glycoproteins, and mucin-like proteins, including MUC1. These interactions may provide protective interfaces that mitigate harm from gastric acidity and bile salts (17, 97). MFGM supplementation enhanced the survival of *Lacticaseibacillus rhamnosus* throughout gastrointestinal transit in animal models and modulated the expression of genes associated with exopolysaccharide (EPS) production. Transmission electron microscopy investigations have revealed direct interactions between MFGM phospholipids and the surfaces of certain *Lactobacillus* strains (98–100). In addition to providing structural protection, MFGM-derived glycans and glycolipids may function as metabolic substrates for particular probiotic species, facilitating bacterial proliferation in carbohydrate-limited environments (8, 16). These data indicate that MFGM may serve not only as a physical carrier but also as a *biologically active interface* that facilitates probiotic persistence and metabolic activity. However, the molecular mechanisms underlying MFGM–probiotic interactions remain incompletely understood. Emerging evidence indicates that the polar lipid fraction of MFGM, which includes phospholipids, sphingomyelin, and gangliosides, may modulate microbial colonization and mucosal immune signaling (17, 49). These processes could, in turn, contribute to gut–brain axis communication during early life. MFGM has significant potential as a natural encapsulation matrix for probiotic delivery; nevertheless, further research is required to clarify the molecular interactions at play and to assess their impact on probiotic efficacy in the human gastrointestinal tract.

### 4.2 The effects of milk fat globule membrane on infant health

**Figure 2:**
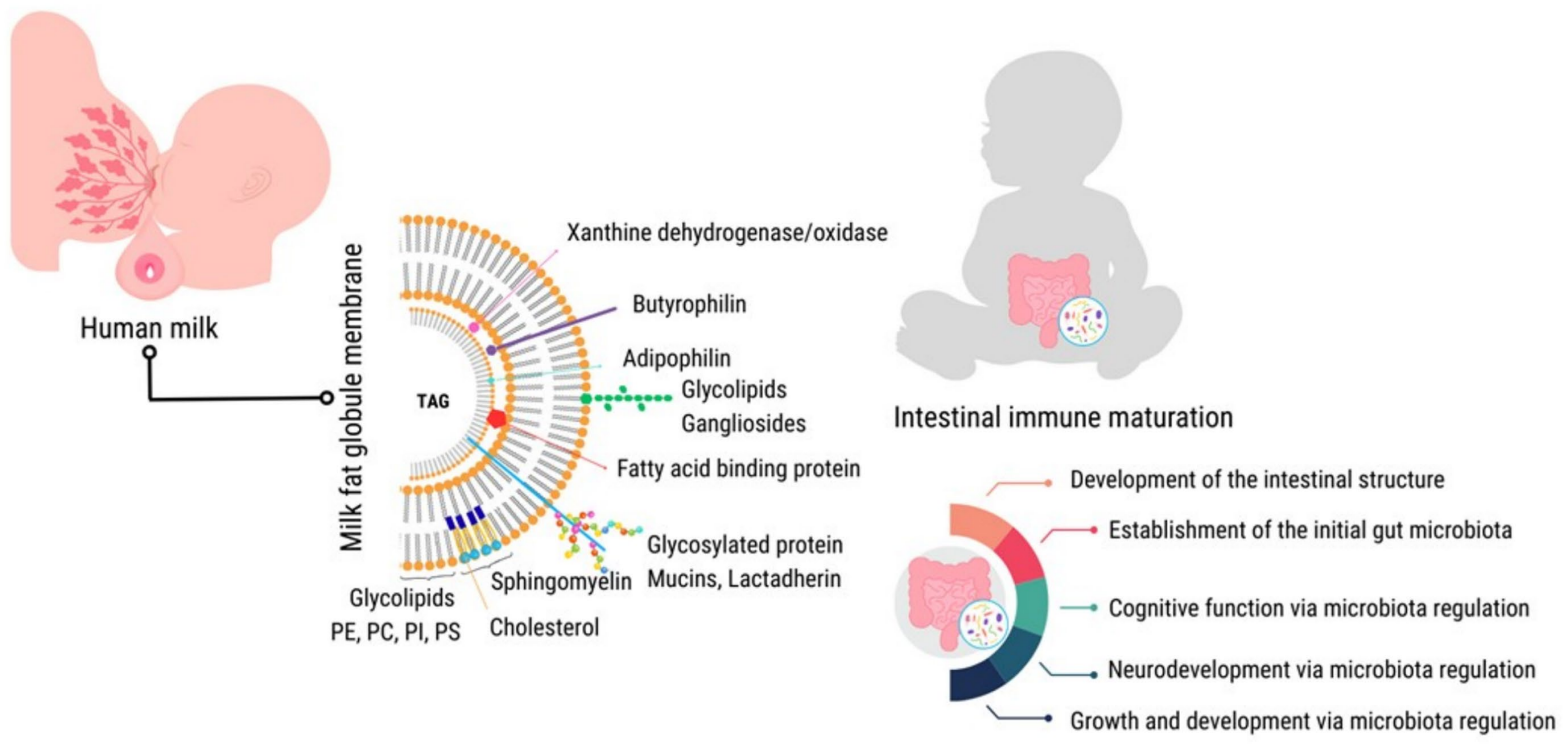
MFGM as a nutritional modulator of gut microbiota and health in infants [adapted from Yadav et al. (64) and Mohamed et al. (86)]. MFGM is enriched in polar lipids, glycolipids, cholesterol, and membrane-specific proteins that support intestinal immune maturation by promoting intestinal development, establishing the gut microbiota, and regulating cognitive, neurodevelopmental, and growth outcomes. Created using Canva.

MFGM is a complex membrane structure surrounding milk fat droplets and contains PLs, sphingolipids, glycoproteins, gangliosides, and various other biologically active components (9, 36). Studies have shown that MFGM exerts significant immunomodulatory effects (101), supports neurological development (102), and contributes to the maturation of the intestinal mucosal barrier (103) during infancy. These health benefits are primarily attributed to its modulatory impact on the gut microbiota (39, 104–111). Microbiota development in the first months after birth plays an important role in shaping lifelong health and disease susceptibility (112, 113). Microbiota-mediated methods through which MFGM may impact infant development and health outcomes have therefore received more study. In this context, the effects of MFGM on microbiota development and its contribution to infant health through these effects have been investigated (39, 106–111, 114–116). The subsequent sections highlight the significance of MFGM in influencing early microbial colonization and the resultant health implications of these microbiota-mediated interactions. Figure 2 illustrates the impact of MFGM on the intestinal microbiota and related health outcomes in infants.

## 5 Microbiota-mediated effects of milk fat globule membrane on early-life gut microbiota

During infancy, the gastrointestinal microbiota develops under the influence of many factors, such as environmental exposures, mode of delivery, antibiotic use, and diet (112, 113, 117). The composition and maturation of the gut microbiota are influenced by MFGM, as evidenced by reduced *Escherichia coli* and increased *Bifidobacterium* levels in the stools of infants fed a ganglioside-supplemented formula, suggesting that MFGM plays a modulatory role in shaping gut microbiota during early life (118). MFGM was found to interact specifically with probiotics such as *Lactobacillus* and *Bifidobacterium*. Thereby increased the resistance of probiotics to bile salts (98, 119). MFGM increased *Lactobacillus rhamnosus* GG survival during transit through the murine gastrointestinal tract (98). MFGM also enabled probiotics to adhere more efficiently to the intestinal epithelium. Strain-specific increased adhesion of *Lactobacillus* and *Bifidobacterium* was observed, along with a significant reduction in pathogen colonization. Compared to the control group, supplementation with MFGs under culture conditions resulted in a more diverse mucin–phospholipid milieu, promoting enhanced probiotic adhesion to the intestinal epithelium (99, 120). The early-life gut forms a dynamic microbial community. In which MFGM components—phospholipids, gangliosides, and glycoproteins—provide nutrients and attachment sites for beneficial bacteria such as *Lactobacillus* and *Bifidobacterium*.

These components also block binding sites and modulate immune responses, thereby limiting the growth of harmful bacteria. Through these actions, MFGM directly promotes a stable, healthy gut microbiome early in life. These data indicate that MFGM may influence early microbial ecosystems by fostering beneficial taxa and restricting pathogen colonization, thereby establishing a microbial environment conducive to immunological and metabolic development throughout infancy.

### 5.1 *In vitro* evidence

In the *in vitro* study conducted by Raz et al., experiments with *Bacillus subtilis* demonstrated that MFG size is a determining factor in bacterial behavior. Small MFGs (~2.3 μm) increased bacterial growth by approximately 100-fold, while large MFGs (~7 μm) promoted the formation of complex biofilm structures, particularly by inducing the expression of the tapA operon (among the most important indicators of biofilm formation) (121). Yadav et al. isolated two probiotic strains, *Lactiplantibacillus plantarum* MRK3 and *Limosilactobacillus fermentum* MK1, from infant feces and investigated the effect of human milk MFGM on these probiotics. In an *in vitro infant digestion model involving Lactiplantibacillus plantarum* MRK3 and human milk *MFGM*, probiotic survival was increased, and MRK3 was physically integrated into the human milk MFGM matrix during digestion. Combining human milk MFGM and the probiotic MRK3 strain increased cell viability by preventing H2O2-induced / *E. coli*-induced toxic effects in Caco-2 intestinal epithelial cells. This study demonstrates that human milk MFGM may support the integrity and survival of probiotic bacteria as they are transported to the intestines, thanks to its membrane matrix and phospholipids (122). MFGM may affect the initial formation of microbial communities in the infant gut by improving probiotic survival and epithelial adherence. Alterations in microbiota may subsequently influence the formation of the intestinal barrier, immunological signaling pathways, and metabolic activities throughout early development.

### 5.2 Preclinical animal evidence

In various animal models and human studies, MFGM supplementation has been shown to provide systemic benefits. These include increased antioxidant enzyme activities (SOD, CAT, GSH-Px), decreased inflammatory markers (IL-1β, IL-6, TNF-α, LPS), and strengthened intestinal barrier integrity (increased tight junction proteins, decreased LPS levels) (Supplementary Table 1) (104, 105, 115, 123). At the microbiota level, the relative abundance of beneficial bacteria, including probiotic species such as *Lactobacillus, Bifidobacterium, Akkermansia*, and *Faecalibaculum,* increases, while potential pathogens (such as *Escherichia-Shigella* and *Enterococcus*) decrease, supporting microbial balance. Furthermore, in animals and human infants receiving MFGM supplements, the *Firmicutes/Bacteroidetes* ratio is regulated, SCFA production is increased, and intestinal microbiota diversity is positively affected (104, 114–116, 123, 124). The results of the studies can be summarized under three main headings: the effects of MFGM on lipid metabolism (1, 114, 115), microbiota modulation (114, 115, 124), and intestinal barrier function and immune effects (104, 105, 115). Current data suggest that MFGM components regulate gut microbiota composition and metabolic activity. These changes then affect intestinal barrier integrity, immune signaling pathways, and overall metabolic responses. The effects of MFGM on microbiota may shape larger developmental outcomes, such as immune system maturation, intestinal function, and possibly neurodevelopment in early life.

### 5.3 Human clinical evidence

In human infants, have been shown that MFGM-containing formulas increase microbiota diversity, particularly promoting the proliferation of beneficial species such as *Bifidobacterium bifidum* and *Akkermansi*a (125). These findings suggest that MFGM may have potential beneficial effects on the healthy development of early-life microbiota and support immune and metabolic functions. However, the limited number of clinical studies, heterogeneous doses, combined interventions, small and variable sample sizes, different intervention durations, and heterogeneous study designs restrict the generalizability of these findings (*in vivo* neonatal rodent or sows models and randomized controlled human infant studies with control groups receiving standard formula, breast milk, or emulsions). Consequently, standardized, controlled, and comprehensive studies are needed to more accurately assess the effects of MFGM. Current evidence suggests that the MFGM may affect infant health by altering gut bacteria. Its active components seem to help early bacterial growth, strengthen the gut lining, and support immune system development in infants. More well-designed studies are needed to learn how much these bacteria-related effects impact growth, metabolism, and brain development.

## 6 Microbiota-mediated health benefits of milk fat globule membrane

### 6.1 Microbiota-related immune and intestinal growth

Dietary factors and microbial colonization during infancy shape the mucosal immune system, which supports health in later life (126). The capacity of MFGM to modulate microbiota has important implications, especially on the development of the immune system, intestinal mucosal barrier, and gastrointestinal tract (48, 124, 127). These data indicate that MFGM may affect immune and intestinal development through microbiota-linked pathways. Its bioactive components help beneficial microbes colonize the gut and regulate host–microbe interactions. These actions may support mucosal immune responses and strengthen the intestinal barrier in early life. Proteomic and phosphoproteomic analyses of proteins isolated from MFGM revealed that these structures are actively involved in various signaling pathways regulating immune responses. In the study, proteins showing changes in expression and phosphorylation levels based on comparative proteomic and phosphoproteomic analyses include perilipin-2, xanthine dehydrogenase/oxidase, glycosylation-dependent cell adhesion molecule 1, fatty acid synthase, clusterin, heat shock protein β-1, β-casein, αS1-casein, and αS2-casein, fatty acid synthase, and sodium/nucleoside cotransporter. The majority of MFGM proteins found in bovine colostrum and mature milk are associated with cell adhesion and innate immune responses, including Fc gamma R-mediated phagocytosis, African trypanosomiasis, B cell receptor signaling pathways, *Staphylococcus aureus* infection, and *Yersinia* infection (128). In a study conducted by Christensen et al. on healthy term infants, the effects of infant formula enriched with bovine milk-derived MFGM on the gut microbiota, short-chain fatty acids, and immune biomarkers were investigated. In infants fed the bovine MFGM-enriched formula, the composition and diversity of the fecal microbiota were positively affected compared to those fed standard formula, and an increase in the levels of short-chain fatty acids was observed. Additionally, it was noted that MFGM supplementation may have positive regulatory effects on gastrointestinal tolerance and intestinal immune function (116). In the study by Ortega-Anaya et al., *Lactobacillus* interacted directly with phospholipids from the MFGM, increasing the bacterial cell surface electronegativity either by adsorption onto the cell envelope or by incorporating into the phospholipid molecules in the cell (97). MFGM supplementation supported normal neonatal intestinal development, restored microbiota composition closer to a healthy state, and strengthened the mucosal barrier (129, 130). Regarding intestinal development, MFGM supplementation protected the intestinal mucosa in neonatal and adult animals, decreased apoptosis rates, and increased the activity of epithelial defense genes such as MUC1, MUC2, and the Reg3 gene family. Especially in models of intestinal damage such as short bowel syndrome and necrotizing enterocolitis, MFGM contributed to the reconstruction of the intestinal epithelium (124, 129–134) (Supplementary Table 2). It also contributed to a reduced incidence of infection (135). In the study conducted by Bhinder et al., it was demonstrated that intestinal development normalized in rat pups fed MFGM-supplemented formula, intestinal epithelial cell proliferation and tight junction protein development increased, and the microbiota became more similar to that of breastfed pups. Furthermore, MFGM provided protection against colitis associated with intrarectal exposure to *Clostridioides difficile* toxins in rats (129).

**Figure 3:**
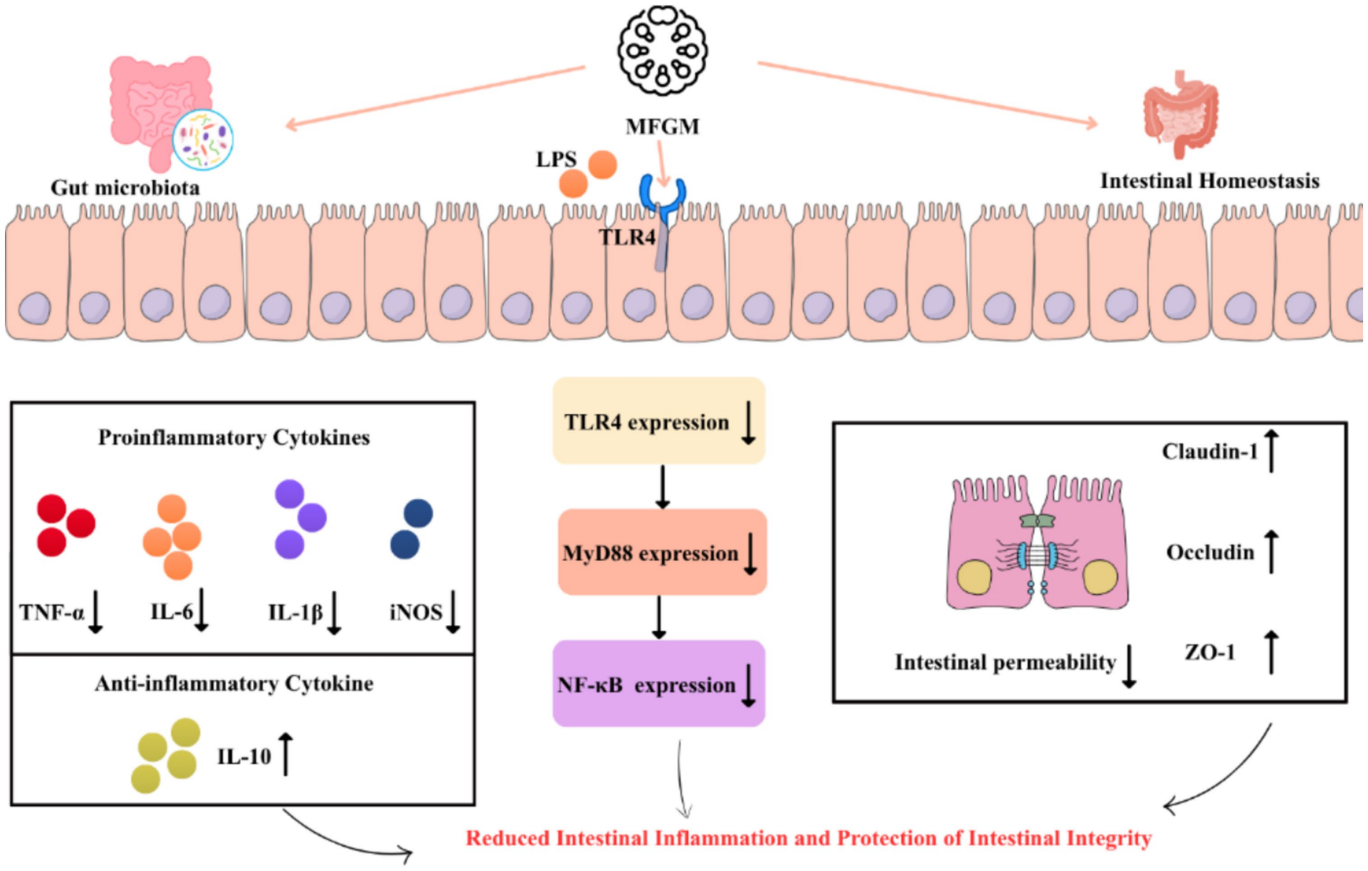
Proposed mechanisms underlying the protective effects of milk fat globule membrane (MFGM) on intestinal inflammation and barrier integrity (↑: increase, ↓: decrease). Created using Canva.

MFGM supplementation suppressed proinflammatory cytokines (TNF-α, IL-1β, IL-6), elevated anti-inflammatory IL-10, and increased gene expression of tight junction proteins such as claudin-1, occludin, and ZO-1, which support the barrier integrity of the intestine, particularly in inflammation-induced models involving LPS or DSS (124, 129–134, 136). In MFGM-fed groups, intestinal permeability was reduced, and relative mRNA expression levels of immune-related genes (TLR2, TLR4, NLRP3, and Caspase-1) were suppressed in the ileum (131, 134). At the same time, MFGM has beneficial effects on neonatal rats with NEC by suppressing inflammation via the TLR4/MyD88/NF-κB pathway. TLR4 is an important component of the inflammatory response in NEC, and MFGM reduced the ileal expression of TLR4, mRNA expression of IL-1β, IL-6, iNOS, and TNF-α in rats with NEC. To further elucidate the inflammatory mechanism of MFGM, MFGM treatment in LPS-stimulated intestinal epithelial cells-6 partially inhibited the increase in TLR4, MyD88, and p-NF-κBp65 protein levels and reduced the nuclear translocation of NF-κBp65 (61). These findings indicate that MFGM modulates the inflammatory response at the protein level and limits NF-κB-mediated gene transcription. It is conceivable that MFGM may protect intestinal integrity by modulating inflammatory signaling at both the protein expression and transcriptional levels (133). The decrease in mRNA levels for TLR2 and TLR4 may be consistent with the suppression of the TLR4–MyD88–NF-κB pathway, as pathway activity depends not only on transcript abundance but also on the receptor’s surface protein level, the activity of adapter proteins (e.g., MyD88), the phosphorylation of NF-κB p65, and its translocation to the nucleus (131, 133). Figure 3 provides a general overview of the mechanism by which MFGM affects intestinal inflammation and barrier integrity.

In summary, it has been demonstrated that MFGM can reduce intestinal inflammation by modulating the TLR4/MyD88/NF-κB signaling pathway. It has been observed that MFGM reduces TLR4 activation, suppresses MyD88-dependent signaling, inhibits NF-κB phosphorylation and nuclear translocation, and consequently reduces the expression of pro-inflammatory cytokines such as TNF-α, IL-1β, and IL-6. However, further studies are needed to evaluate the reduction in TLR2 and TLR4 mRNA levels. Additionally, it has been suggested that MFGM may enhance intestinal barrier integrity by increasing the expression of tight junction proteins such as occludin, claudin-1, and ZO-1. The findings also suggest that MFGM may influence brain development through the microbiota–gut–brain axis, via changes in the gut microbiota that affect the immune system, metabolism, and early brain development.

### 6.2 Microbiota–gut–brain axis and neurodevelopment

As part of this work, a structured literature search was carried out in PubMed, Web of Science, and Scopus using predefined keyword groups (e.g., “infant,” “MFGM,” “MFGM-related compounds,” “neurodevelopment,” “cognitive function,” “microbiota,” “clinical study,” and “preclinical study”). The retrieved publications were screened and classified according to study type and area of focus. Among the relevant literature, three original clinical studies focusing on microbiota and neurodevelopment were found (39, 107, 108), along with two original preclinical investigations (106, 110), which are presented in Supplementary Table 3. Several studies in this field have investigated the effects of MFGM on neurodevelopment, behavior, and cognitive development (38, 137–139), as well as on the gut microbiota (109, 125). The accumulating data indicate that the impact of MFGM on neurodevelopment may be partially mediated by interactions within the microbiota–gut–brain axis. Alterations in microbial composition and metabolites may affect immunological signaling, metabolic pathways, and brain development in early life.

Preclinical studies have been designed using controlled animal models to investigate the potential effects of MFGM and its associated components on neurodevelopmental processes. In this area, some studies have focused explicitly on the effects of MFGM on either neurodevelopment or the gut microbiota (110, 111). In their preclinical study, Mudd et al. (110) investigated the effects of a diet enriched with prebiotics, milk fat globule membrane, and lactoferrin in piglets from postnatal day 2 to 31. Magnetic resonance imaging (MRI) assessments between days 21 and 31 revealed no significant differences in total brain volume or the relative volumes of 19 brain regions compared to controls (110). Nieto-Ruiz et al. (111) evaluated infants from the COGNIS randomized controlled trial who were fed breast milk, standard formula, or an MFGM- and LC-PUFA-enriched formula for 18 months. The enriched formula group showed favorable growth outcomes and significant improvements in cortical visual evoked potentials, suggesting enhanced visual cortex development linked to early nutritional intervention (111). This COGNIS RCT indicated that an enriched formula did not hinder growth or early general neurodevelopment, and, for visual cortical maturation, it could more closely resemble breast milk compared with standard formula. While breast milk was the optimal source of nourishment, formula enriched with bioactive components may help close the neurodevelopmental gap in formula-fed infants. However, because the formula contained multiple bioactive components, including MFGM and LC-PUFAs, the specific contribution of MFGM to these effects cannot be clearly distinguished.

In the study by Zhao et al. (n = 44), researchers compared the effects of breastfeeding, standard formula, and a fortified formula enriched with prebiotics, MFGM, and lactoferrin on gut microbiota composition at one month of age. Alpha-diversity (Simpson and Shannon indices) was highest in the breastfed group, while beta-diversity analyses showed distinct microbial profiles between breastfed and formula-fed infants. Notably, *Bifidobacterium* abundance was significantly higher in the breastfed group (29.89%) compared to the common (2.66%) and fortified formula groups (2.27%). Key MFGM components—lactadherin, sialic acid, and phospholipids—were positively associated with *Bifidobacterium* levels and inversely associated with potentially pathogenic genera such as *Veillonella* and *Escherichia/Shigella*. Gene enrichment analysis identified upregulation of beneficial metabolic pathways in *Bifidobacterium*. These findings suggest that MFGM components may contribute to a more favorable gut microbiota profile in early infancy, although they may not fully replicate the effects of breastfeeding (106). Two studies examined how MFGM supplementation affects infant gut microbiota and metabolomic profiles. MFGM-containing formulas were associated with reduced amino acid metabolites, indicating altered microbial fermentation (109). Additionally, an increase in Bacteroides species was observed (125), though metabolomic changes did not reach statistical significance after correction (109, 125).

Notably, four long-term clinical studies have investigated the impact of MFGM on neurodevelopment and cognitive and behavioral outcomes (38, 137–139). In the COGNIS study series, the development of children who were fed breast milk, standard formula, or a formula enriched with MFGM, LC-PUFAs, and synbiotics during the first 18 months of life was evaluated at ages 2.5 and 4 years (137, 138). Among infants fed the enriched formula for 18 months, follow-up assessments up to 2.5 years of age revealed reduced behavioral problems, particularly improvements in affective and externalizing behaviors. The results indicated that this group was comparable to breastfed children in terms of behavioral outcomes. In a subsequent analysis evaluating longer-term effects, significant improvements in language development were observed at age 4, suggesting that MFGM may contribute positively to neurodevelopmental processes (138). The investigations indicated that a formula with MFGM, LC-PUFA, and synbiotics was associated with fewer behavioral problems up to 2.5 years of age and enhanced language development at 4 years compared with a conventional formula. The enhanced formula more closely resembled breast milk in numerous respects. Nonetheless, due to the intervention including multiple bioactive components, the observed advantages are probably attributable to their synergistic effects rather than to MFGM in isolation. Moreover, variables including maternal IQ, education, and socioeconomic status may potentially affect neurodevelopmental outcomes. Consequently, the current evidence does not permit precise isolation of the specific contribution of MFGM to these outcomes.

In another study series, infants fed a formula containing MFGM and LC-PUFAs for 12 months showed increased total brain volume and improved white matter integrity as assessed by MRI. These findings were interpreted as a positive contribution to early brain development (139). In the same cohort, which was followed up to 6.5 years of age, MFGM supplementation was suggested to enhance myelination and support the development of early cognitive skills (38).

Formulas enriched with MFGM appear to confer certain neurodevelopmental advantages in infancy and early childhood when compared to standard formulas. Several randomized controlled trials (RCTs) have reported improvements in specific neurocognitive domains in infants fed MFGM-fortified formulas. For instance, Nieto-Ruiz et al. (39) demonstrated that children in the enriched formula group showed higher IQ and vocabulary scores at age 6 compared to the breastfed group, along with greater cortical thickness and parietal lobe volume (39). These neuroanatomical findings were associated with improved neuropsychological test outcomes. Similarly, Chen et al. (108) reported that by 4 months, infants in the MFGM-fortified formula group achieved motor development scores comparable to breastfed peers, and both groups outperformed the standard formula group (108). In addition to behavioral and cognitive outcomes, MRI-based assessments in piglet models (110) showed no significant differences in relative brain volume, yet provided a valuable translational perspective, emphasizing the complexity of neurodevelopmental metrics beyond gross brain morphology. Furthermore, a longitudinal RCT showed that infants with faster microbial maturation trajectories—promoted by enriched formula—achieved higher cognitive and expressive language scores at 12 months, although these differences leveled off by age 4 (107). Collectively, these findings suggest that MFGM may support early-life brain development through both direct nutritional mechanisms and gut–brain axis modulation. These observations support a framework in which bioactive components derived from MFGM interact with intestinal microbiota, influencing microbial metabolites, immune signaling pathways, and neural development, thus contributing to cognitive and behavioral outcomes in early childhood.

Despite promising findings, studies investigating the neurodevelopmental impact of MFGM-enriched formulas exhibit several methodological and interpretative limitations. First, the heterogeneity in neurodevelopmental assessment tools—ranging from early motor scores to advanced cognitive testing and MRI-based measures—limits comparability across studies. For example, while Chen et al. (108) used short-term developmental scales within the first 6 months, Nieto-Ruiz et al. (39) employed neuroimaging and cognitive tests at age 6, making longitudinal synthesis challenging. Moreover, variations in intervention components (e.g., MFGM combined with LC-PUFAs, synbiotics, or OPO) make it difficult to isolate the specific effect of MFGM on brain development. Many studies also lack baseline assessments of neurodevelopment, limiting causal interpretation. Finally, sample sizes in some trials were relatively small, and few accounted for confounding variables such as parental education, home environment, or early stimulation, which are critical determinants of neurocognitive outcomes.

### 6.3 Growth and metabolic development associated with microbiota

The effects of MFGM supplementation on infant growth parameters have generally been comparable to those of standard formula and breastfeeding, with some favorable trends. In a large-scale RCT, Li et al. (132) found no significant differences in weight or length gain among infants receiving MFGM- or probiotic-enriched formulas compared with those receiving standard formulas (140). However, breastfed infants exhibited greater early weight gain until approximately four months of age. Notably, from 5 to 12 months, infants fed the F19 formula showed a slight but statistically significant increase in weight gain relative to the breastfed group. In another study, Best et al. (135) demonstrated that MFGM-enriched formula enhanced fat-free mass and total body volume compared with breastfeeding, indicating a potential advantage in lean tissue accretion (135).

While Chen et al. (108) observed no significant group differences in anthropometric outcomes such as head circumference, height, and weight, enriched formula-fed infants exhibited faster overall growth trends in the early months (108). Additionally, selected gut microbial genera (e.g., *Sutterella*, *Methanobrevibacter*) were linked to head circumference and bone mineral density, suggesting potential indirect effects of gut modulation on physical development. These findings suggest that gut bacteria may help link MFGM intake to infant growth and metabolism. MFGM-induced changes in gut microbial composition and fermentation activity may affect nutrient intake, metabolic signaling, and growth-related physiological processes in growing infants. These findings collectively imply that while MFGM may not markedly alter growth trajectories, it contributes to qualitative improvements in body composition and functional developmental markers. In this randomized controlled trial (n = 108), children who received MFGM-, LC-PUFA-, and synbiotic-enriched formula from birth to 18 months were assessed at six years of age alongside standard formula-fed and breastfed peers. While no group differences were found in growth metrics or basic language comprehension, children in the enriched formula group showed higher IQ and vocabulary scores than the breastfed group, and better attention and impulse control than both other groups. MRI results revealed greater parietal lobe volume and increased cortical thickness—particularly in the left occipitotemporal sulcus—in both the enriched formula and breastfed groups, which correlated with better neurocognitive performance (39). These findings suggest that early-life consumption of enriched formulas may lead to measurable structural and functional enhancements in brain development that persist in childhood.

Research exploring the role of MFGM in supporting infant growth and physical development is constrained by several critical gaps. Most notably, anthropometric outcomes such as weight, length, and head circumference have shown minimal or inconsistent differences between enriched and standard formulas, and the clinical significance of observed differences is often unclear. While some studies have incorporated body composition analyses, most rely on basic growth metrics without considering functional development markers such as bone density or lean mass accretion over time. Furthermore, many trials do not extend follow-up beyond the first year of life, leaving the long-term impact of MFGM on growth trajectories poorly understood. The lack of stratified analyses by sex, birth weight, or prematurity status also limits applicability to high-risk subgroups. In addition, inter-study variability in formula composition and energy density further complicates interpretation, as differences in caloric intake may confound the observed growth effects.

## 7 Safety of MFGM supplementation

Although the addition of MFGM supplementation to infant nutrition has been shown to have positive biological and developmental effects, further research is needed on certain safety issues before it can be widely used in infant formulas. Current studies indicate that bovine-derived MFGM is generally well tolerated and does not have an adverse effect on infant growth or gastrointestinal tolerance in randomized controlled trials (19, 116, 141). Although there are few studies on the safety and tolerability of formulas enriched with MFGM, these studies have emphasized that they are safe and well-tolerated and exhibit effects like those of standard formulas (140, 142, 143). However, depending on the source of milk used in the composition of MFGM preparations, as well as the extraction method and processing technologies, significant variations in the content of phospholipids, gangliosides, and membrane proteins may be observed. These factors make it difficult to ensure standardization across studies and limit the comparability of biological activity results (8, 92, 144) (Supplementary Table 4). Another important consideration is that structural changes resulting from processing can affect the activity of MFGM. High-temperature treatments, homogenization, and drying processes can lead to denaturation of membrane proteins and disruption of the structural integrity of bioactive lipids (46, 145–148). Furthermore, given the presence of bovine-derived protein components, the potential for allergenicity cannot be completely ruled out. MFGM has a complex protein structure and contains various protein components; therefore, it has been noted that protein-related immunological effects must be carefully evaluated (149–153). Another important consideration is the lack of standardized international recommendations regarding the optimal dose and composition of MFGM in infant formulas (41, 154, 155). Multi-component formulations containing LC-PUFAs, lactoferrin, or synbiotics in addition to MFGM have been used; this limits the ability to observe the effect of MFGM alone (39, 107, 125). Future studies should focus on standardized production methods, quality control, long-term safety monitoring, and mechanistic evaluation of isolated MFGM fractions to support evidence-based practice in infant nutrition.

## 8 Conclusion and future directions

This review synthesizes current evidence on the role of the MFGM in infant nutrition. Evidence links MFGM, alone or with other bioactive components, to improvements in infant brain development, cognitive outcomes, gut microbiota balance, and immune maturation. However, studies often do not isolate the unique impact of MFGM alone, making its specific role unclear.

The existing evidence has many limitations. In many clinical studies, MFGM is combined with other compounds, such as LC-PUFAs, synbiotics, or lactoferrin. This makes it hard to see what benefits come from MFGM alone. There is considerable variability across studies in intervention duration, participant demographics, and formula composition. Dosage details are difficult to interpret because MFGM-containing substances are part of age-adjusted formulas rather than given separately. Moreover, MFGM supplementation is predominantly found in specific or premium infant formulae, introducing diversity to the existing data. Collectively, these limitations constrain the capacity to ascertain solid conclusions about the exact role of MFGM in infant health outcomes.

Randomized controlled trials with subsequent follow-up have begun to address these questions. A recent assessment discovered just seven original papers, comprising five randomized controlled trials, underscoring the paucity of clinical data now available. While current research indicates that MFGM-containing formulas may benefit certain aspects of infant health, the extent and causes of these effects remain unclear. Future research needs to emphasize meticulously designed, adequately powered randomized controlled trials using standardized formulations and explicitly specified intervention regimens. It is essential to directly examine MFGM’s independent effects by testing treatments where MFGM is given alone, not with other bioactive components. This approach clarifies MFGM’s specific biological functions and informs evidence-based recommendations for infant nutrition.

## Glossary

ADPH: Adipophilin
BTN: Butyrophilin
CD36: Cluster of Differentiation 36
DAG: Diacylglycerol
ER: Endoplasmic Reticulum
FABP: Fatty Acid Binding Protein
FFA: Free Fatty Acid
MAG: Monoacylglycerol
MFG: Milk Fat Globules
MFGM: Milk Fat Globule Membrane
MUC: Mucin
PC: Phosphatidylcholine
PE: Phosphatidylethanolamine
PAS 6/7: Periodic Acid Schiff 6/7
PI: Phosphatidylinositol
PLs: Phospholipids
PP3: Proteose Peptone 3
PS: Phosphatidylserine
RCT: Randomized Controlled Trial
TAG: Triacylglycerol
