# Supplementary material for: Milk fat globule membrane in early-life nutrition: composition, production, and biological effects on infant immune maturation, intestinal development, neurocognitive function, and growth
Source: Front Nutr. 2026 Jun 18;13:1851487. doi: 10.3389/fnut.2026.1851487 (PMC13323632; doi:10.3389/fnut.2026.1851487)
Supplement: Supplementary file 1 [file Table_1.DOCX]

Supplementary Material

**Table 1.** Milk Fat Globule Membrane and Early-Life Microbiota Development: Evidence from Preclinical and Clinical Studies

| **Population** | **Interventions** | **Outcomes measured and key findings** | **References** |
| --- | --- | --- | --- |
| BALB/c male mice | Group 1: Control (saline)  Group 2: MFGM supplementation (400mg/kg BW)  Duration: 18 weeks | Group 2 vs Group 1:   - Exhaustive swimming time ↑ (antifatigue effect) - Blood glucose ↑ - Blood lactic acid ↓ - Lactate dehydrogenase ↓ - Antioxidant enzymes (SOD, CAT, GSH-Px) ↑ - MDA ↓ - Nrf2 protein expression ↑ - Caspase-3 expression ↓ - Relative abundance of *Bacteroides*, *Butyricimonas*, and *Anaerostipes* ↑ - Relative abundance of Verrucomicrobiota ↑ | (1) |
| C57BL/6 mice offspring exposed to a maternal high-fat diet | Group 1: Control diet (CD) offspring  Group 2: Maternal high-fat diet offspring (HFD)  Group 3: HFD + neonatal MFGM supplementation (1000 mg/kg BW/day) during suckling (PD3–PD21)  *Table 1 (continued)* | Group 3 vs Group 2:   - Serum TG ↓ - Fasting blood glucose ↓ - AST and ALT ↓ - Glucose tolerance ↑ - Gut microbiota modulation: ↑ *Parabacteroides*, *Bifidobacterium*, *Faecalibaculum*, *Lactobacillus* (male offspring) - ↑ *Parabacteroides*, *Alistipes* (female offspring) | (2) |
| C57BL/6J male mice (DSS-induced colitis model) | Group 1: Control (PBS)  Group 2: MFGM (50 mg/kg BW/day)  Group 3: DSS (4% DSS)  Group 4: MFGM + DSS  Duration: 4 weeks MFGM pretreatment + 7 days DSS | Group 4 vs Group 3:   - Colon length ↑ and histological damage ↓ - Pro-inflammatory cytokines IL-1β and IL-6 ↓, IL-10 ↑ - Mucus barrier genes MUC2, MUC4, Reg3b, and Reg3g ↑ - Beneficial microbiota genera *Faecalibaculum* and *Roseburia* ↑ - Hepatic injury markers AST and ALT ↓ - Hepatic oxidative stress ↓ (MDA ↓, CAT/SOD/GSH-Px ↑) | (3) |
| Sprague–Dawley rats (offspring of obese dams fed a high-fat diet during pregnancy and lactation) | Group 1: Control diet  Group 2: Control diet + MFGM (400 mg/kg BW)  Group 3: High-fat diet (HFD)  Group 4: HFD + MFGM (400 mg/kg BW) | Group 4 vs Group 3:   - Gut microbiota composition improved - Relative abundance of *Lactobacillus* ↑ and *Akkermansia* ↑ - Relative abundance of *Escherichia*-*Shigella* ↓ and *Enterococcus* ↓ - Serum inflammatory markers LPS, IL-1β, IL-6, and TNF-α ↓ - Brain inflammatory markers and microglial activation (Iba1) ↓ | (4) |
| Sprague-Dawley female rats (maternal obesity model induced by high-fat diet during pregnancy and lactation)  *Table 1 (continued)* | Group 1: Control diet  Group 2: Control diet + MFGM-PL (400 mg/kg BW/day)  Group 3: High-fat diet (HFD)  Group 4: HFD + MFGM-PL (400 mg/kg BW/day) | Group 4 vs Group 3:   - Serum TG, LDL-C, leptin, IL-1β, IL-6, TNF-α and LPS ↓ - HDL-C ↑ - Gut microbiota diversity and richness ↑ - *Bacteroidetes*/*Firmicutes* ratio ↑ - Relative abundance of *Akkermansia* ↑ and *Ruminococcaceae* ↓ - Insulin signaling pathways (PI3K/Akt) in liver, adipose tissue, and skeletal muscle ↑ - Glucose tolerance and insulin sensitivity improved | (5) |
| Sprague–Dawley male rats (short-bowel syndrome model) | Group 1: Sham surgery  Group 2: SBS (massive small-bowel resection)  Group 3: SBS + MFGM (1.5 g/kg BW/day) for 14 days | Group 2 vs Group 1:   - Intestinal permeability ↑ - Crypt depth and goblet cell number ↓ - MUC1 and MUC2 positive cells ↓ - Firmicutes abundance decreased and the acetate concentration ↑ - IL-1β and Caspase-1 expression ↑ indicating intestinal barrier impairment and inflammation   Group 3 vs Group 2:   - Intestinal permeability ↓ - Crypt depth and goblet cell number ↑ - MUC1 and MUC2 positive cells ↑ - Firmicutes abundance increased, and the acetate concentration ↓ - NLRP6 and IL-18 expressions increased while IL-1β and Caspase-1 expressions decreased, indicating improved colonic mucus barrier and reduced inflammation | (6) |
| Large White and Landrace pregnant sows and their neonatal piglets  *Table 1 (continued)* | Group 1: CON (basal diet)  Group 2: MFGM supplementation (9.9 g/day) from gestation day 85 until farrowing | Group 2 vs Group 1:   - Plasma parameters of sows improved with increased ALB, LDL-C, and NEFA concentrations - Umbilical cord blood of piglets showed higher GH, IgA, and glucose levels - Jejunal villus height increased, and crypt depth in the duodenum and jejunum - Expression of tight junction genes ↑ (Occludin, Claudin-1, Claudin-2, Claudin-4, ZO-1), mucins ↑ (Mucin-2, Mucin-4, Mucin-13) and immune-related genes ↑ (TNF-α, IFN-γ, IL-22, TLR2, TLR4) - Fecal microbiota diversity in piglets increased with higher abundance of Christensenellaceae_R-7_group and *Alloprevotella* | (7) |
| Healthy infants  (7-18 days old) | Group 1: Control infant formula  Group 2: Formula + bovine MFGM  (5 g/L)  Group 3: Human milk  60-day feeding period | Group 2 vs Group 1:   - Higher stool butyrate, total branched-chain fatty acids, isovalerate, and lactate concentrations - Modest changes in stool microbiota composition, including increased *Akkermansia* and *Bacteroides* taxa   Group 2 vs Group 3:   - Higher stool acetate, propionate, and total SCFA levels - *Bifidobacterium bifidum* ↑ and *B. catenulatum* ↑   Group 1 vs Group 3:   - Higher stool pH in the control formula group compared with human milk - No significant differences were detected between groups in oral microbiota diversity or stool immune biomarkers (α-defensin, β-defensin, calprotectin, sIgA) | (8) |
| Late preterm infants (34–36⁺⁶ weeks gestation), appropriate for gestational age | Group 1: Nutrient-enriched formula (NEF) containing higher protein, vitamin D, butyrate, and bovine MFGM (22 kcal/30 ml)  Group 2: Standard term formula (STF) (20 kcal/30 ml)  Reference group: Breastfeeding infants (BFR)  Intervention duration: from enrollment until 120 days corrected age  *Table 1 (continued)* | - No significant differences in fecal microbiota diversity, richness, or total bacterial load between the randomized formula groups at 60 and 120 days of age. | (9) |
| Healthy infants | Group 1 Breastfed infants (BF)  Group 2 Standard formula (SF)  Group 3 MFGM-supplemented formula (MFGM-enriched whey protein fraction, 5 g/L) from 21 ±7 days to 4 months | Group 3 vs Group 2:   - Serum insulinogenic amino acids and protein degradation metabolites ↓ Fatty acid oxidation and ketogenesis ↑ - Metabolic profile shifted toward BF-like metabolism - No significant differences in fecal microbiota composition - Microbial diversity progression moderated   Group 3 vs Group 1:   - Metabolome remained distinct from BF infants but showed partial convergence toward BF metabolic phenotype - Fecal microbiota composition still differed from that of BF infants | (10) |
| Healthy infants | Group 1 Breastfed infants (BF)  Group 2 Standard formula (SF)  Group 3 Experimental formula supplemented with bovine MFGM (EF) (~2-6 months) | Group 3 vs Group 2:   - Lower fecal concentrations of several amino acids and amino acid degradation products (ornithine, isoleucine, glutamate, phenylalanine, tyrosine, valine, glycine) - Lower microbial metabolites, including lactate and succinate - Reduced markers of amino acid fermentation - Fecal water content ↓   Group 3 vs Group 1:   - Fecal microbiota composition remained more similar to SF than BF infants - Overall impact of MFGM on microbiota composition was moderate, but changes in fecal metabolome indicated altered microbial activity | (11) |
| Healthy term infants | Group 1 Control infant formula  Group 2 Formula supplemented with bovine MFGM + lactoferrin | Group 2 vs Group 1:   - Subtle differences in gut microbiota β-diversity at 4 months - *Bacteroides plebeius* ↑ and *Bacteroides uniformis* ↑ - Metabolite profile differences, including lower medium-chain fatty acids, deoxycarnitine, and glycochenodeoxycholate - Some carbohydrates and steroid metabolites increased - Overall stool microbiome composition remained broadly similar between groups | (12) |

Abbreviations: BF, breast feeding; CAT, catalase; DCX, doublecortin; EF, experimental formula; GSH-Px, glutathione peroxidase; HDL-C, high density lipoprotein cholesterol; HFD, high fat diet; IF, infant formula; IL, interleukin; LDL-C, low density lipoprotein cholesterol; LPS, lipopolysaccharide; MDA, malondialdehyde; MFGM, milk fat globule membran; MUC, mucin; SBS, short bowel syndrome; SF, standart formula; SOD, superoxide dismutase; TG; triglyceride; TNF-α, tumour necrosis factor alpha

**References**

1. Zou X, Yokoyama W, Liu X, Wang K, Hong H, Luo Y, et al. Milk Fat Globule Membrane Relieves Fatigue via Regulation of Oxidative Stress and Gut Microbiota in BALB/c Mice. Antioxidants (Basel, Switzerland). 2023;12(3).

2. Ye L, Zhang Q, Xin F, Cao B, Qian L, Dong Y. Neonatal Milk Fat Globule Membrane Supplementation During Breastfeeding Ameliorates the Deleterious Effects of Maternal High-Fat Diet on Metabolism and Modulates Gut Microbiota in Adult Mice Offspring in a Sex-Specific Way. Front Cell Infect Microbiol. 2021;11:621957.

3. Wu Z, Liu X, Huang S, Li T, Zhang X, Pang J, et al. Milk Fat Globule Membrane Attenuates Acute Colitis and Secondary Liver Injury by Improving the Mucus Barrier and Regulating the Gut Microbiota. Frontiers in immunology. 2022;13:865273.

4. Yuan Q, Gong H, Du M, Li T, Mao X. Milk fat globule membrane supplementation to obese rats during pregnancy and lactation promotes neurodevelopment in offspring via modulating gut microbiota. Frontiers in nutrition. 2022;9:945052.

5. Li T, Yuan Q, Gong H, Du M, Mao X. Gut microbiota mediates the alleviative effect of polar lipids-enriched milk fat globule membrane on obesity-induced glucose metabolism disorders in peripheral tissues in rat dams. International journal of obesity (2005). 2022;46(4):793-801.

6. Yu Z, Li Y, Niu Y, Tang Q, Wu J. Milk Fat Globule Membrane Enhances Colonic-Mucus-Barrier Function in a Rat Model of Short-Bowel Syndrome. JPEN Journal of parenteral and enteral nutrition. 2021;45(5):916-25.

7. Zhang X, Wu Y, Ye H, Feng C, Han D, Tao S, et al. Dietary milk fat globule membrane supplementation during late gestation increased the growth of neonatal piglets by improving their plasma parameters, intestinal barriers, and fecal microbiota. RSC advances. 2020;10(29):16987-98.

8. Christensen C, Kok CR, Harris CL, Moore N, Wampler JL, Zhuang W, et al. Microbiota, metabolic profiles and immune biomarkers in infants receiving formula with added bovine milk fat globule membrane: a randomized, controlled trial. Frontiers in nutrition. 2024;11:1465174.

9. Best KP, Yelland LN, Collins CT, McPhee AJ, Rogers GB, Choo J, et al. Growth of late preterm infants fed nutrient-enriched formula to 120 days corrected age-A randomized controlled trial. Front Pediatr. 2023;11:1146089.

10. Lee H, Slupsky CM, Heckmann AB, Christensen B, Peng Y, Li X, et al. Milk Fat Globule Membrane as a Modulator of Infant Metabolism and Gut Microbiota: A Formula Supplement Narrowing the Metabolic Differences between Breastfed and Formula-Fed Infants. Mol Nutr Food Res. 2021;65(3):e2000603.

11. He X, Parenti M, Grip T, Lönnerdal B, Timby N, Domellöf M, et al. Fecal microbiome and metabolome of infants fed bovine MFGM supplemented formula or standard formula with breast-fed infants as reference: a randomized controlled trial. Scientific reports. 2019;9(1):11589.

12. Chichlowski M, Bokulich N, Harris CL, Wampler JL, Li F, Berseth CL, et al. Effect of bovine milk fat globule membrane and lactoferrin in infant formula on gut microbiome and metabolome at 4 months of age. Current Developments in Nutrition. 2021;5(5):nzab027.
