# Supplementary material for: Milk fat globule membrane in early-life nutrition: composition, production, and biological effects on infant immune maturation, intestinal development, neurocognitive function, and growth
Source: Front Nutr. 2026 Jun 18;13:1851487. doi: 10.3389/fnut.2026.1851487 (PMC13323632; doi:10.3389/fnut.2026.1851487)
Supplement: Supplementary file 2 [file Table_2.DOCX]

Supplementary Material

**Table 2.** Milk Fat Globule Membrane and Its Impact on Immune and Intestinal Development: Insights from Preclinical and Clinical Research

| **Population** | **Interventions** | **Outcomes measured and key findings** | **References** |
| --- | --- | --- | --- |
| Caco-2/Goblet intestinal cell model | Group 1: Control *Lactobacillus* strains  Group 2: *Lactobacillus* strains treated with MFGM-derived phospholipids (MPL) | Group 2 vs Group 1:   - Enhanced adherence of *Lactobacillus casei* and specifically *Lactobacillus delbrueckii* to intestinal epithelial cells - Increased bacterial surface electronegativity - MPL modified bacterial cell envelope properties and adhesion kinetics - Enhanced potential probiotic colonization capacity | (1) |
| C57BL/6J mice | Group 1: Control group  Group 2: MFGM (50 mg/kg BW/day)  Group 3: DSS (4% DSS-induced colitis)  Group 4: MFGM + DSS | Group 4 vs Group 3:   - Colitis severity ↓ (DAI, histological damage) - Pro-inflammatory cytokines ↓; IL-10 ↑ - Mucosal barrier gene expression ↑ (MUC2, MUC4, Reg3b, Reg3g) - Beneficial gut microbiota (*Faecalibaculum*, *Roseburia*) ↑ - Hepatic injury and oxidative stress ↓ | (2) |
| Sprague-Dawley male rats | Group 1: Sham  Group 2: SBS  Group 3: SBS+MFGM (1.5 g/kg/d) | Group 3 vs Group 2:   - MPO-positive inflammatory cells in liver tissue ↓ - Hepatic expression of NLRP3 inflammasome markers (NLRP3, ASC, Caspase-1, IL-1β) ↓ | (3) |
| Neonatal Sprague-Dawley rats | Group 1: Breast-fed  Group 2: Formula fed (FF)  Group 3: FF+MFGM6 (6 g/L)  Group 4: FF+MFGM12 (12 g/L) | Group 4 vs Group 2:   - NEC incidence ↓ (85% → 46.7%) - Survival rate ↑ (30% → 66.7%) - Intestinal histological injury ↓ - Oxidative stress ↓ (MDA ↓, SOD ↑) - Enterocyte apoptosis ↓ and proliferation ↑ - Tight-junction protein expression (claudin-1) ↑ - TLR4 expression ↓ and inflammatory cytokines (IL-1β, IL-6, TNF-α, iNOS) ↓ | (4) |
| Male Sprague-Dawley rats  *Table 2 (continued)* | Group 1: Sham surgery  Group 2: SBS (massive small-bowel resection)  Group 3: SBS + MFGM (1.5 g/kg/day gavage) | Group 3 vs Group 2:   - Intestinal permeability ↓ (FD-40 levels) - Bacterial translocation to MLN and liver ↓ - Tight junction proteins (claudin-1, claudin-2, occludin) ↑ - MUC1 expression ↑ - NLRP3 inflammasome activation ↓ (NLRP3, ASC, caspase-1 ↓) - IL-1β ↓ and IL-18 ↑   Group 3 vs Group 1:   - No significant improvement in body weight or villus height | (5) |
| Low-birth-weight C57BL/6J female neonatal mice | Group 1: Control group  Group 2: LPS group  Group 3: MFGM100 (100 mg/kg BW) + LPS  Group 4: MFGM200 (200 mg/kg BW) + LPS  (Postnatal day 4-21) | Group 3 vs Group 2:   - Intestinal histological injury ↓ - Plasma pro-inflammatory cytokines (TNF-α, IL-6, IL-1β) ↓ - Antioxidant activity ↑ (SOD, CAT) - Tight junction gene expression ↑ (ZO-1, occludin, claudin-1) - Mucin gene expression ↑ (MUC1, MUC2) - TLR2 and TLR4 expression ↓   Group 4 vs Group 2:   - Similar anti-inflammatory and barrier-protective effects (generally weaker than 100 mg/kg dose) | (6) |
| Male Wistar rats (9 weeks old), infected with *Listeria monocytogenes* | Group 1: Skimmed milk diet (low MFGM)  Group 2: Sweet buttermilk diet (high MFGM) | Group 2 vs Group 1:   - Fecal excretion of *Listeria monocytogenes*↓ - Luminal *Listeria* counts in proximal small intestine, caecum, and colon ↓ - *Listeria* colonization of intestinal mucosa ↓ - Translocation of *Listeria* to the spleen and liver ↓ - Gastric listericidal activity ↑ - Inhibition of *Listeria* adhesion to intestinal epithelial cells (Caco-2 model) ↑ | (7) |
| BALB/c male mice  *Table 2 (continued)* | Group 1: Control diet (corn oil) + saline  Group 2: Control diet + LPS  Group 3: Milk fat diet (anhydrous milk fat + 10% MFGM) + saline  Group 4: Milk fat diet + LPS  Duration: 5 weeks of feeding before the LPS challenge | Group 4 vs Group 2:   - Gut permeability ↓ - Serum inflammatory cytokines ↓ (IL-6, IL-10, IL-17, MCP-1, IFN-γ, TNF-α, IL-3) - Systemic inflammatory response attenuated   Additional observation:   - LPS-treated mice fed the control diet showed markedly higher intestinal permeability (~1.8-fold) and stronger inflammatory responses | (8) |
| Fischer-344 male rats | Group 1: Control group (corn oil)  Group 2: Anhydrous milk fat (AMF)  Group 3: AMF + MFGM group  (13 weeks) | **Group 3 vs Group 1:**   - Aberrant crypt foci (ACF) incidence ↓ - Colon preneoplastic lesions ↓   Group 3 vs Group 2:   - Lower number of aberrant crypt foci - Reduced colon carcinogenesis markers | (9) |
| Neonatal Sprague-Dawley rats | Group 1: Breast milk  Group 2: Control formula  Group 3: Formula supplemented with MFGM (6 g/L)  Postnatal days 5–15 | Group 3 vs Group 1:   - Intestinal development parameters were largely comparable to those observed in breast-fed animals   Group 3 vs Group 2:   - Villus height in the jejunum and ileum ↑ - Crypt depth in the jejunum, ileum, and colon ↑, proliferation marker Ki-67 in the jejunum and colon ↑ - Tight junction proteins Claudin-3 and Claudin-4 ↑ - Paneth and Goblet cell numbers ↑ - MUC2 expression in the ileum and colon ↑, indicating improved intestinal development and barrier maturation | (10) |
| Large White and Landrace pregnant sows and their piglets | Group 1: Control group  Group 2: MFGM (9.9 g/day) during late gestation (day 85 until farrowing) | Group 2 vs Group 1:  Maternal outcomes (sows):   - Plasma albumin (ALB), LDL-C, and NEFA ↑ - Fecal SCFA concentrations (acetate, propionate, butyrate) ↑ - Relative abundance of *Prevotella*   Offspring outcomes (piglets):   - Umbilical cord blood: GH, IgA, GLU, and NEFA ↑ - Intestinal morphology: jejunal villus height ↑; duodenal and jejunal crypt depth ↓ - Barrier-related genes: Occludin, Claudin-1, Claudin-2, Claudin-4, and ZO-1 ↑ - Mucin genes: MUC2, MUC4, and MUC13 ↑ - Immune-related genes: TNF-α, IFN-γ, IL-22, TLR2, and TLR4 ↑ - Gut microbiota: microbial diversity ↑; Christensenellaceae_R-7_group and *Alloprevotella* ↑ | (11) |
| Healthy infants  (7–18 days of age) | Group 1: Control formula  *Table 2 (continued)*  (cow’s milk-based infant formula)  Group 2: Formula + bovine MFGM  (5 g/L)  Group 3: Human milk  Intervention duration: 60 days | Group 2 vs Group 1:   - Stool butyrate, lactate, and total branched-chain fatty acids ↑ - Isobutyrate and isovalerate ↑ - Higher abundance of *Akkermansia* and *Bacteroides* species   Group 3 vs Group 2:   - Lower stool microbiota diversity in group 3 - Lower acetate, propionate, and total SCFA in group 3   Group 3 vs Group 1:   - Lower stool pH in group 3 - No significant differences in α-defensin, β-defensin, calprotectin, or sIgA between groups | (12) |
| Late preterm infants (34-36⁺⁶ weeks gestation), appropriate for gestational age | Group 1: Nutrient-enriched formula (NEF) containing higher protein, vitamin D, butyrate, and bovine MFGM (22 kcal/30 ml)  Group 2: Standard term formula (STF) (20 kcal/30 ml)  Reference group: Breastfeeding infants (BFR)  Intervention duration: from enrollment until 120 days corrected age | Group 1 vs Group 2:   - Reduced risk of infectious illness in the NEF group | (13) |
| Healthy term infants  (<2 months at enrollment) | Group 1: Standard formula  Group 2: Experimental formula + bovine MFGM  Group 3: Breast-fed reference group | Group 2 vs Group 1:   - Acute otitis media ↓ - Antipyretic use ↓ - Pneumococcal IgG ↓   Group 2 vs Group 3:   - Similar incidence of acute otitis media | (14) |

Abbreviations: ASC, apoptosis-associated speck-like protein containing a CARD; DSS, dextran sulfate sodium; FF, formula fed; IFN-γ, interferon gamma; iNOS, LPS, lipopolysaccharide; MCP, monocyte chemotactic protein; MDA, malondialdehyde; MFGM, milk fat globule membrane; MPO, myeloperoxidase; MUC, mucin; NEF, nutrient-enriched formula; NLRP3, NOD-like receptor protein 3; PLs, phospholipids; SBS, short bowel syndrome; TLR, Toll-like receptor; TNF-α, tumour necrosis factor alpha; ZO-1, zonula occludens-1

*Table 2 (continued)*

**Table 2 (continued)**

**References**

*Table 2 (continued)*

1. Ortega-Anaya J, Marciniak A, Jiménez-Flores R. Milk fat globule membrane phospholipids modify adhesion of Lactobacillus to mucus-producing Caco-2/Goblet cells by altering the cell envelope. Food Research International. 2021;146:110471.

2. Wu Z, Liu X, Huang S, Li T, Zhang X, Pang J, et al. Milk Fat Globule Membrane Attenuates Acute Colitis and Secondary Liver Injury by Improving the Mucus Barrier and Regulating the Gut Microbiota. Frontiers in immunology. 2022;13:865273.

3. Yu Z, Huang S, Li Y, Niu Y, Chen H, Wu J. Milk Fat Globule Membrane Alleviates Short Bowel Syndrome-Associated Liver Injury in Rats Through Inhibiting Autophagy and NLRP3 Inflammasome Activation. Frontiers in nutrition. 2022;9:758762.

4. Zhang D, Wen J, Zhou J, Cai W, Qian L. Milk Fat Globule Membrane Ameliorates Necrotizing Enterocolitis in Neonatal Rats and Suppresses Lipopolysaccharide-Induced Inflammatory Response in IEC-6 Enterocytes. JPEN Journal of parenteral and enteral nutrition. 2019;43(7):863-73.

5. Li Y, Wu J, Niu Y, Chen H, Tang Q, Zhong Y, et al. Milk Fat Globule Membrane Inhibits NLRP3 Inflammasome Activation and Enhances Intestinal Barrier Function in a Rat Model of Short Bowel. JPEN Journal of parenteral and enteral nutrition. 2019;43(5):677-85.

6. Huang S, Wu Z, Liu C, Han D, Feng C, Wang S, et al. Milk Fat Globule Membrane Supplementation Promotes Neonatal Growth and Alleviates Inflammation in Low-Birth-Weight Mice Treated with Lipopolysaccharide. BioMed research international. 2019;2019:4876078.

7. Sprong RC, Hulstein MF, Lambers TT, van der Meer R. Sweet buttermilk intake reduces colonisation and translocation of Listeria monocytogenes in rats by inhibiting mucosal pathogen adherence. The British journal of nutrition. 2012;108(11):2026-33.

8. Snow DR, Ward RE, Olsen A, Jimenez-Flores R, Hintze KJ. Membrane-rich milk fat diet provides protection against gastrointestinal leakiness in mice treated with lipopolysaccharide. Journal of dairy science. 2011;94(5):2201-12.

9. Snow DR, Jimenez-Flores R, Ward RE, Cambell J, Young MJ, Nemere I, et al. Dietary milk fat globule membrane reduces the incidence of aberrant crypt foci in Fischer-344 rats. J Agric Food Chem. 2010;58(4):2157-63.

10. Bhinder G, Allaire JM, Garcia C, Lau JT, Chan JM, Ryz NR, et al. Milk fat globule membrane supplementation in formula modulates the neonatal gut microbiome and normalizes intestinal development. Scientific reports. 2017;7(1):45274.

11. Zhang X, Wu Y, Ye H, Feng C, Han D, Tao S, et al. Dietary milk fat globule membrane supplementation during late gestation increased the growth of neonatal piglets by improving their plasma parameters, intestinal barriers, and fecal microbiota. RSC advances. 2020;10(29):16987-98.

12. Christensen C, Kok CR, Harris CL, Moore N, Wampler JL, Zhuang W, et al. Microbiota, metabolic profiles and immune biomarkers in infants receiving formula with added bovine milk fat globule membrane: a randomized, controlled trial. Frontiers in nutrition. 2024;11:1465174.

13. Best KP, Yelland LN, Collins CT, McPhee AJ, Rogers GB, Choo J, et al. Growth of late preterm infants fed nutrient-enriched formula to 120 days corrected age-A randomized controlled trial. Front Pediatr. 2023;11:1146089.

14. Timby N, Hernell O, Vaarala O, Melin M, Lönnerdal B, Domellöf M. Infections in infants fed formula supplemented with bovine milk fat globule membranes. J Pediatr Gastroenterol Nutr. 2015;60(3):384-9.
