# Supplementary material for: Milk fat globule membrane in early-life nutrition: composition, production, and biological effects on infant immune maturation, intestinal development, neurocognitive function, and growth
Source: Front Nutr. 2026 Jun 18;13:1851487. doi: 10.3389/fnut.2026.1851487 (PMC13323632; doi:10.3389/fnut.2026.1851487)
Supplement: Supplementary file 3 [file Table_3.DOCX]

Supplementary Material

**Table 3.** The Role of MFGM-Enriched Nutrition in Neurodevelopment, Growth, and Development: Preclinical and Clinical Perspectives

| **Population** | **Interventions** | **Outcomes measured and key findings** | **References** |
| --- | --- | --- | --- |
| Healthy term infants, 1-month-old | Group 1: Breastfeeding  Group 2: Common formula  Group3: Modified formula supplemented with MFGM components | Group 2 and Group 3 vs Group 1:   - Lower height and weight at birth and at 1 month   Group 3 vs Group 2:   - No significant differences in growth rate or other anthropometric and clinical parameters at 1 month | (1) |
| Yorkshire Piglet | Group 1: Control diet  Group 2: Test diet (prebiotics + lactoferrin + MFGM) | Group 2 vs Group 1:   - Lower radial diffusivity and mean diffusivity in the internal capsule indicating more advanced white matter maturation - Regional differences in gray and white matter tissue concentrations detected by voxel-based morphometry - No differences in total brain volume or relative volumes of 19 brain regions. No differences in spatial learning performance, although the latency to choice was longer in the test group | (2) |
| Healthy full-term infants | Group 1 Breast milk (BMG) Group 2 Regular formula (RFG) Group 3 Fortified formula containing OPO and MFGM (FFG)  Follow-up: 1, 4, and 6 months | Group 3 vs Group 1:   - Neurodevelopment scores (gross motor and developmental quotient) closer to breastfed infants - Growth parameters comparable between groups | (3) |
| Healthy full-term infants | Group 1 Standard formula (SF) Group 2 Experimental formula (EF: synbiotics + LC-PUFA + MFGM)  Group 3 Breastfed (BF) | Group 2 vs Group 1:   - Neurodevelopment scores at 12 months were comparable, but EF infants with faster microbiota maturation showed higher language and expressive language scores - Growth parameters (weight, length, head circumference) were similar between groups   Group 2 vs Group 3:   - Neurodevelopmental outcomes at 12 months and 4 years were similar to those of breastfed infants - Growth and development patterns were comparable to those of breastfed infants | (4) |
| *Table 3 (continued)*  Healthy children (COGNIS study, n=108, assessed at 6 years) | Group 1: Standard formula (SF) Group 2: Experimental formula enriched with MFGM + LC-PUFAs + synbiotics (EF) Group 3: Breastfed (BF) | Group 2 vs Group 1:   - EF children showed greater parietal brain volume (right parietal region, postcentral gyrus and precuneus) and greater cortical thickness in several brain areas including the occipito-temporal sulcus and insular cortex - EF children had higher vocabulary and IQ scores in the K-BIT test   Group 2 vs Group 3:   - EF children showed higher IQ and vocabulary scores compared with BF children - EF children had greater left orbital cortex volüme - Growth parameters, including BMI, head circumference and waist circumference at 6 years, did not differ between groups | (5) |
| Late preterm infants (34-36⁺⁶ weeks gestation), appropriate for gestational age | Group 1: Nutrient-enriched formula (NEF) containing higher protein, vitamin D, butyrate, and bovine MFGM (22 kcal/30 ml)  Group 2: Standard term formula (STF) (20 kcal/30 ml)  Reference group: Breastfeeding infants (BFR)  Intervention duration: from enrollment until 120 days corrected age | Group 1 vs Group 2:   - No significant difference in weight gain rate (29.8 vs 28.0 g/day) - No significant differences in length gain or head circumference gain   Group 1 vs Reference (breastfed):   - Higher fat-free mass, body mass, and body volume at 120 days | (6) |
|  | Group 1 Standard formula (SF) Group 2 Formula + bovine MFGM Group 3 Formula + probiotic F19  Reference group Breastfed infants | Group 2 vs Group 1:   - Weight gain ↔ - Length gain ↔ - Head circumference gain ↔   Group 2 vs Breastfed:   - Growth parameters generally comparable after the early months   Group 3 vs Group 1:   - Weight gain ↔ - Length gain ↔ - Head circumference gain ↔ | (7) |

# Abbreviations: AMD, adjusted mean differences; IQ, intelligence quotient; LC-PUFA, long-chain polyunsaturated fatty acids; MFGM, milk

*Table 3 (continued)*

# fat globule membrane; MRI, magnetic resonance imaging; RCT, randomized controlled trial; OPO, 1,3-dioleoyl-2-palmitoylglycer

# References

1. Zhao J, Yi W, Liu B, Dai Y, Jiang T, Chen S, et al. MFGM components promote gut Bifidobacterium growth in infant and in vitro. European Journal of Nutrition. 2022:1-12.

2. Mudd AT, Alexander LS, Berding K, Waworuntu RV, Berg BM, Donovan SM, et al. Dietary prebiotics, milk fat globule membrane, and lactoferrin affects structural neurodevelopment in the young piglet. Frontiers in pediatrics. 2016;4:4.

3. Chen B, Jia Q, Chen Z, You Y, Liu Y, Zhao J, et al. Comparative evaluation of enriched formula milk powder with OPO and MFGM vs. breastfeeding and regular formula milk powder in full-term infants: A comprehensive study on gut microbiota, neurodevelopment, and growth. Food & Function. 2024;15(3):1417-30.

4. Cerdó T, Ruíz A, Acuna I, Nieto-Ruiz A, Diéguez E, Sepúlveda-Valbuena N, et al. A synbiotics, long chain polyunsaturated fatty acids, and milk fat globule membranes supplemented formula modulates microbiota maturation and neurodevelopment. Clinical Nutrition. 2022;41(8):1697-711.

5. Nieto-Ruiz A, García-Santos JA, Verdejo-Román J, Diéguez E, Sepúlveda-Valbuena N, Herrmann F, et al. Infant formula supplemented with milk fat globule membrane, long-chain polyunsaturated fatty acids, and synbiotics is associated with neurocognitive function and brain structure of healthy children aged 6 years: the COGNIS study. Frontiers in nutrition. 2022;9:820224.

6. Best KP, Yelland LN, Collins CT, McPhee AJ, Rogers GB, Choo J, et al. Growth of late preterm infants fed nutrient-enriched formula to 120 days corrected age-A randomized controlled trial. Front Pediatr. 2023;11:1146089.

7. Li X, Peng Y, Li Z, Christensen B, Heckmann AB, Stenlund H, et al. Feeding infants formula with probiotics or milk fat globule membrane: a double-blind, randomized controlled trial. Frontiers in Pediatrics. 2019;7:347.
