# Supplementary material for: Milk fat globule membrane in early-life nutrition: composition, production, and biological effects on infant immune maturation, intestinal development, neurocognitive function, and growth
Source: Front Nutr. 2026 Jun 18;13:1851487. doi: 10.3389/fnut.2026.1851487 (PMC13323632; doi:10.3389/fnut.2026.1851487)
Supplement: Supplementary file 4 [file Table_4.DOCX]

Supplementary Material

**Table 4.** Comparative Major Lipid and Protein Characteristics of MFGM Derived from Different Sources

| **Source** | **Major Lipids** | **Major Proteins** | **References** |
| --- | --- | --- | --- |
| Human milk-derived MFGM | Sphingomyelin  Phosphatidylcholine  Phosphatidylethanolamine  Gangliosides | Mucin 1 (MUC 1), Xanthine Oxidoreductase (XDH/XO or XOR), Butyrophilin (BTN), Lactadherin (PAS 6/7, MFG-E8), CD 36, Adipophilin, and Fatty acid-binding protein (FABP) | (1) |
| Bovine milk-derived MFGM | Phosphatidylcholine  Phosphatidylethanolamine Sphingomyelin | Butyrophilin, Xanthine Oxidoreductase, Adipophilin, PAS 6/7 (Lactadherin/MFG-E8) | (2) |
| Yak milk-derived MFGM | Phosphatidylcholine  Phosphatidylethanolamine  Sphingomyelin | Butyrophilin, Xanthine Oxidase/dehydrogenase, Adipophilin | (3) |
| Buttermilk-derived MFGM | Phosphatidylcholine,  Phosphatidylethanolamine, Sphingomyelin | Butyrophilin, Xanthine Oxidoreductase, Adipophilin | (4) |

# Abbreviations: MFGM, milk fat globule membrane

# References

1. Thum C, Wall C, Day L, Szeto IMY, Li F, Yan Y, et al. Changes in Human Milk Fat Globule Composition Throughout Lactation: A Review. Frontiers in nutrition. 2022;9:835856.

2. Fong BY, Norris CS, MacGibbon AKH. Protein and lipid composition of bovine milk-fat-globule membrane. International Dairy Journal. 2007;17(4):275-88.

3. Luo J, Huang Z, Liu H, Zhang Y, Ren F. Yak milk fat globules from the Qinghai-Tibetan Plateau: Membrane lipid composition and morphological properties. Food chemistry. 2018;245:731-7.

4. Astaire JC, Ward R, German JB, Jiménez-Flores R. Concentration of Polar MFGM Lipids from Buttermilk by Microfiltration and Supercritical Fluid Extraction. Journal of dairy science. 2003;86(7):2297-307.
